# Explaining the unexpected COVID-19 trends and potential impact across Africa.

**DOI:** 10.12688/f1000research.74363.1

**Published:** 2021-11-22

**Authors:** Daniel Oduro-Mensah, Ebenezer Oduro-Mensah, Peter Quashie, Gordon Awandare, Laud Okine

**Affiliations:** 1 Department of Biochemistry, Cell and Molecular Biology, College of Basic and Applied Sciences, University of Ghana, Accra, Ghana; 2 West African Center for Cell Biology of Infectious Pathogens, College of Basic and Applied Sciences, University of Ghana, Accra, Ghana; 3 Ga-East Municipal Hospital, Ghana Health Service, Accra, WY1895, Ghana; 4 Noguchi Memorial Institute for Medical Research, College of Health Sciences, University of Ghana, Accra, LG 581, Ghana

**Keywords:** COVID-19, Immunobiography, Inflamm-aging, Inflammation, Long COVID

## Abstract

Official COVID-19 case counts and mortality rates across Africa are lower than had been anticipated. Research reports, however, indicate far higher exposure rates than the official counts in some countries. Particularly in Western and Central Africa, where mortality rates are disproportionately lower than the rest of the continent, this occurrence may be due to immune response adaptations resulting from (1) frequent exposure to certain pro-inflammatory pathogens, and (2) a prevalence of low-grade inflammation coupled with peculiar modifications to the immune response based on one’s immunobiography. We suggest that the two factors lead to a situation where post infection, there is a rapid ramp-up of innate immune responses, enough to induce effective defense and protection against plethora pathogens. Alongside current efforts at procuring and distributing vaccines, we draw attention to the need for work towards appreciating the impact of the apparently widespread, asymptomatic SARS-CoV-2 infections on Africa’s populations *vis a vis* systemic inflammation status and long-term consequences for public health.

## List of abbreviations

COVID: coronavirus disease

LGI: low-grade inflammation

PASC: post-acute sequelae of COVID-19

SARS-CoV-2: severe acute respiratory syndrome coronavirus 2

## Introduction

**Figure 1.**
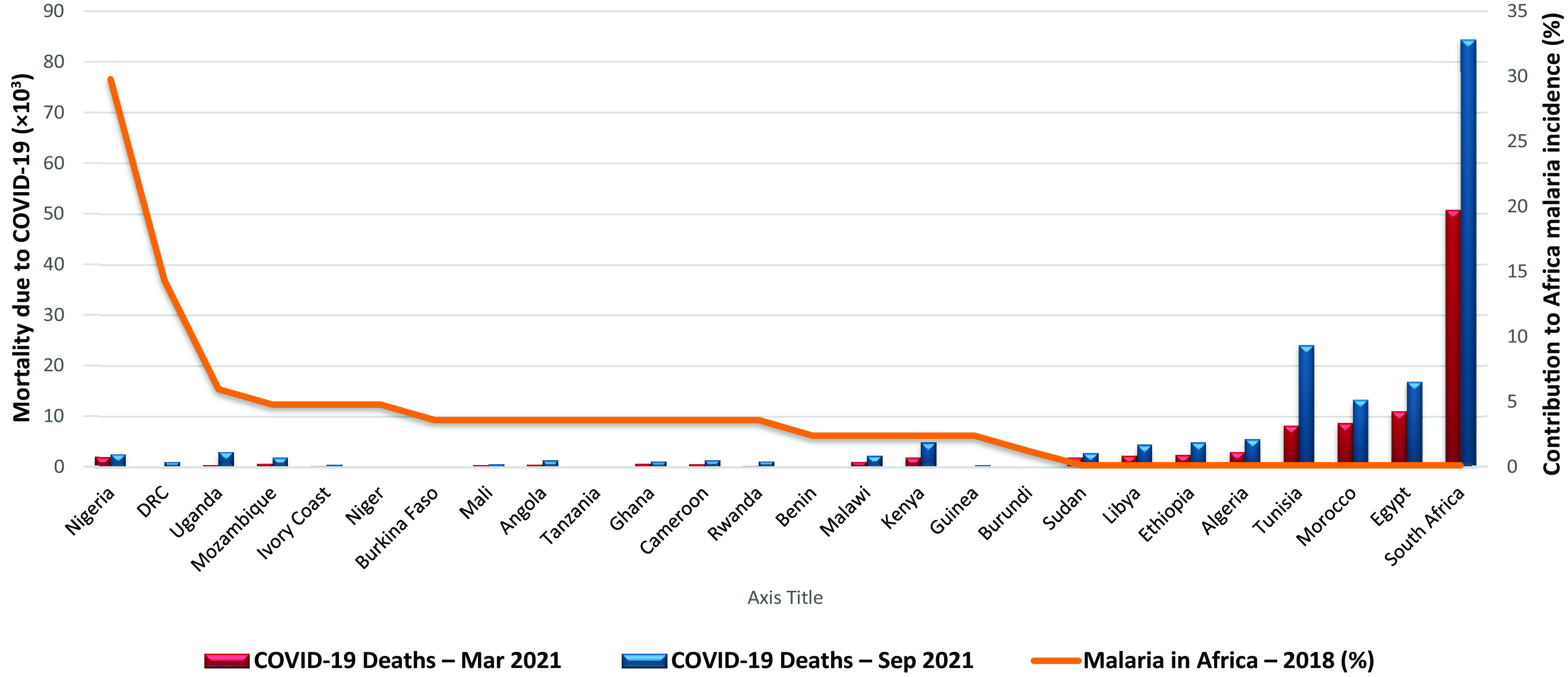
Overlap between COVID-19-related mortality and occurrence of malaria across Africa. Sources: https://www.statista.com/, https://covid19.who.int/; Accessed September 8, 2021.

Despite predictions of being among the worst affected globally, the trajectory of COVID-19 in Africa has been radically different, with far lower morbidity and mortality figures than recorded in the United Kingdom, India, Brazil, the Americas, and across Europe. ^1^ By July 2021, Africa contributed approximately 2 % of global COVID-19 case reports. ^2^ Considering Africa’s relatively ill-resourced healthcare settings, mainly across sub-Saharan Africa, it seems to follow that COVID-19 patients who require hospital admission are disproportionately more likely to die. ^3^ However, Africa’s 16.72 % share of the global population ^4^ contributed only up to 3.5 % of global COVID-19 mortality by July 2021. ^1^^,^^5^ From December 2020 when emerging SARS-CoV-2 variants fueled second and third waves of COVID-19 across the globe ^6^^–^^11^ from December 2020, Africa’s case reports declined steadily from January to mid-May 2021, climbed up sharply and started another decline at the end of June 2021. ^1^^,^^12^^–^^14^ Interestingly among Africa’s five regions, countries in Western and Central Africa appear to be least affected by the COVID-19 pandemic ( Figure 1). ^15^ As of September 2021, the two regions which make up 43.5 % of Africa’s 1.4 billion population ^16^ contributed only 13 % of Africa’s COVID-19 morbidity and 8 % of mortality figures.

Attempts at explaining Africa’s trends include suggestions that not enough testing has been done in the region, ^17^^,^^18^ that Africa has a relatively young population, ^19^^,^^20^ the systematic use of antimalarials in parts of Africa ^21^ and that the third-world conditions across most of the region may mean that people have previously been exposed to viruses molecularly similar to SARS-CoV-2. ^22^^–^^25^ Others have suggested that exposure to similarly inflammatory conditions/pathogens may have led to a situation where individuals have adapted to the effects of inflammation and so are less sensitive to the SARS-CoV-2-induced inflammatory events which are the main drivers of COVID-19 pathogenesis. ^26^^–^^29^ Here, ^1^ we discuss evidence in support of the modification of the immune response due to frequent exposure to inflammatory pathogens, citing malaria, and ^2^ present a second reason to explain why most parts of Africa have been relatively spared the worst of COVID-19.

### Frequent exposure to malaria

Approximately 85 % of global malaria incidence is in Africa. ^30^ Of Africa’s five regions, Western and Central Africa are reported to have the highest malaria incidence, contributing up to 71 % of Africa’s total. In 2017, an inverse relationship between the pro-inflammatory response and exposure to malaria was reported. ^31^ Contrastingly, naïve individuals exhibited a more pro-inflammatory response with higher circulating levels of pro-inflammatory immune mediators. ^32^^,^^33^ Individuals residing in malaria endemic zones often harbor *Plasmodium* spp. infections but are clinically immune. ^34^^,^^35^ Pro-inflammatory mediators are important in expressing the clinical presentation of malaria. It has been suggested that repeated episodes of febrile malaria alter the immune response, resulting in a blunted inflammatory response which shows low levels of pro-inflammatory cytokines and appears to be dominated by anti-inflammatory cytokines. ^36^ This likely plays a significant role in the apparent immunologic tolerance manifesting as clinical immunity to malaria. ^34^^,^^35^^,^^37^ In addition, there appears to be upregulation of certain components of the innate immune response, including phagocytes and interferon-gamma, in frequently exposed individuals. ^36^^,^^38^^,^^39^ We suggest that this altered immune response, characterized by low pro-inflammatory mediator levels, high anti-inflammatory mediator levels and up-regulated innate immune components, offers some protection against SARS-CoV-2-induced inflammation.

In individuals with the altered response due to frequent malaria exposure, SARS-CoV-2 infection may be met by a heightened innate response which overwhelms the reported blocking of some innate immune response pathways by the virus. ^40^^–^^44^ Our submission, therefore, is that although this heightened innate response is limiting to the virus, it does not elicit enough of a pro-inflammatory adaptive response which could lead to the classical COVID-19-associated hyperinflammation. Consequently, the result of SARS-CoV-2-induced inflammation is not the cytokine ‘storm’ that occurs in severe COVID-19, but a milder cytokine ‘drizzle’ that minimizes the deleterious effects of the inflammatory response. This is contrary suggestions, including that by Kusi *et al.,* ^27^ that the innate response may be negatively associated with the observed immunopathology of COVID-19. We maintain that viral load would effectively be suppressed by the innate response, consistent with suggestions by Stertz and Hale, ^43^ with a slow ramp-up in inflammatory cytokine production due to the blunted pro-inflammatory adaptive response. Therefore, patients would only experience mild COVID-19 symptoms, or even have asymptomatic infections. A study on patients in China with asymptomatic COVID-19 revealed that they had relatively lower levels of serum alanine aminotransferase (ALA) and C-reactive protein. ^45^ Previously, it has been shown that ALA may correlate negatively with T cell and natural killer cell activity. ^46^ This supports the suggestion that in individuals with asymptomatic infection, there may be a sharper cell-mediated innate response which significantly moderates the progression to a pro-inflammatory, adaptive immune response.

It is striking how patterns of malaria endemicity appear to contrast COVID-19 mortality patterns in several places across Africa. Countries with the least malaria are the most affected by COVID-related mortality ( Figure 1). Only one (Nigeria) of the top ten malaria endemic countries in Africa was in the top ten of country reports on Africa’s COVID-19 mortality rates as of September 2021. This is likely to be because Nigeria has Africa’s largest population by far. Outside Africa, using Brazil as an example, only one of the top ten malaria endemic areas, Goias, was in the country’s top ten COVID-19 mortality count, at 8th place as of September 2021. ^47^^,^^48^ Meanwhile, Brazil was the world’s second most affected country by COVID-19 mortality at the time. ^49^

In addition to *Plasmodium* spp., other pro-inflammatory pathogens including helminths and human coronaviruses as may be common and/or endemic in many parts of Africa could potentially have similar effects. ^27^^,^^50^^–^^52^ It is unclear, however, whether their effects may be as protective as the malaria effect we allude to. Human coronaviruses (HCoV), for example, has been suggested to play a role in the apparent protection from SARS-CoV-2 due to observed cross-reactivity between HCoV-exposd sera and SARS-CoV-2 antigen. ^25^^,^^53^ However, a contrasting observation suggests that pre-exposure to certain HCoV variants correlates positively with severity of COVID-19. ^54^

### Low-grade inflammation

Low-grade inflammation (LGI) is a state of persistent, low level systemic inflammation marked by approximately 2–4-fold increases in circulating immune pro-inflammatory markers. ^55^^–^^58^ LGI may be due to chronic exposure to stimulatory environmental and lifestyle factors including stress, asymptomatic infections, bad oral hygiene practices, bad diet, obesity, traumatic injury, sedentary behavior, and smoke inhalation. ^59^^–^^61^ The third world living conditions across much of Africa have long fueled a suspicion that LGI might be a relatively widespread phenomenon. Recently, this suspicion has been backed by the increasing reports of full-blown chronic inflammatory diseases among Africa’s populations. ^62^^,^^63^ In individuals with LGI, changes in the immune response may include (1) reduced macrophage function, (2) decreased cytokine production in response to immune challenge, (3) decreased number of naïve T and B cells, (4) altered toll-like receptor expression and signaling, and (5) diminished response to antigen or mitogen stimulation. ^64^ These changes suggest that both the innate and adaptive pro-inflammatory response would be at lower levels relative to individuals without LGI.

At the start of the pandemic, it was predicted that individuals with underlying chronic disease conditions that have systemic inflammation as a common feature would be prone to more severe forms of COVID-19. ^65^^–^^67^ The expectation was that due to the pre-existing immune inflammatory dysfunction, the inflammatory response of such individuals would quickly spiral into hyperinflammation in response to SARS-CoV-2 infection. The situation would be similar for people with low grade inflammation or inflamm-aging but who did not yet have full-blown chronic diseases. ^68^^–^^70^ This, however, appears not to have been the case. Looking at obesity for example, which is a model for LGI, a recent study on patients in America reported that in the first few days after diagnosis, levels of selected inflammation markers were lower in obese COVID-19 patients than in non-obese patients. ^71^ Previous studies on influenza had reported that obese individuals (with LGI) mounted a slow pro-inflammatory response to the respiratory virus, with impaired pathogen-induced and lung-specific responses. ^72^^,^^73^ This impaired adaptive pro-inflammatory response was suggested to contribute to the worse COVID-19 clinical outcomes in obese patients. ^74^^–^^76^ In contrast, we suggest that the delayed response is rather protective, much like in the case of malaria exposure previously described. The worse COVID-19 outcomes in obese patients are more likely found in those with underlying chronic inflammatory diseases ^77^^–^^80^ and are probably due to a combination of factors including extensive dysfunction and dysregulation of the inflammatory response. ^71^^,^^81^^–^^84^ We suggest that, after an inflammatory response is mounted, appropriate metabolic regulation of the response is easily overwhelmed in patients with LGI-related comorbidities. In individuals with only LGI, however, the dysfunction is present but is not so extensive as to have caused organ damage and subsequent chronic disease. Relative to other persons, the already higher circulating levels of inflammatory mediators in LGI individuals feed an immune response, but escalation of the response is impaired. Therefore, there is a slow ramp-up of the pro-inflammatory response coupled with appreciable inflammatory response regulation, a combination which tends to be protective against COVID-19 progression.

Our suggestion is backed by the observation that with the start of vaccination against COVID-19, literature from outside Africa suggest that older adults are less likely to report adverse effects after vaccination. ^85^^–^^87^ Our interpretation of this is that the phenomenon of inflamm-aging, which has characteristics similar to low grade inflammation, ^58^^,^^88^^–^^91^ appears to protect against vaccine side effects. This implies that the combination of higher systemic levels of inflammatory mediators, a blunt pro-inflammatory adaptive immune response, and an apparently impaired innate immune response are useful against COVID-19. In the African population, however, our thinking is that the innate response is not impaired. Rather, as described in the previous section, the history of frequent exposure to pro-inflammatory pathogens leaves the innate response at a higher level relative to individuals from other populations. This may explain why even older Africans appear to have better protection from the virus than older adults from elsewhere. This imprint of immune history on the specific modifications to an individual’s immune response would be consistent with the concept of immunobiography, ^58^^,^^92^ which suggests close links between human health, longevity and individual immune system modifications. ^58^^,^^92^^–^^95^

## Outlook

The focus on COVID-19 characterization and management had been on the clinical presentation of acutely ill patients. Only approximately 14 % of COVID-19 cases are severe enough to require hospital admission. ^96^ References to the 86 % majority who experienced only mild/moderate or asymptomatic COVID-19 usually had to do with the tracking of the infection rate and transmission dynamics. Given the indications that an individual’s systemic inflammation state is altered post SARS CoV-2 infection, there had been the suspicion that even people with asymptomatic infection or mild COVID-19 symptoms would experience some sort of post-acute syndrome. Reports from across the world have indicated such a situation, showing that up to 50 % of non-hospitalized patients who experience mild or moderate COVID-19 continue to have symptoms up to six months and beyond after recovery. ^97^^–^^100^ This has since been termed post-acute sequelae of COVID-19 (PASC). Health issues associated with PASC include fatigue, exercise intolerance, cognitive impairment, anxiety/depression, organ damage, impaired mobility, and reduced quality of life, ^101^^–^^104^ all of which are also associated with LGI and inflamm-aging. ^58^^,^^105^

The prevailing social and environmental conditions across Africa likely mean that the spread of SARS-CoV-2 has been rather extensive. This is supported, for example, by the observation of a 13–27 % average seroprevalence of SARS-CoV-2 antibodies in randomly sampled individuals across Ghana, ^106^ compared to official figures of approximately 0.41 % for cumulative total infection rate as of September 2021. ^107^ Similar situations have been reported in other African countries. ^108^^,^^109^ Interestingly, the 60+ age group showed the highest seropositivity rate 106. Keeping in mind that the antibody test kit used had shown up to 66 % sensitivity and at least 94 % specificity, ^106^ the report suggested that the observation of low sensitivity of antibody test kits may be due to generally low production of antibodies by infected persons rather than a failure of the kits used. The low sensitivity is interesting, particularly when some studies report significant cross-reactivity between other human coronaviruses antibodies and SARS-CoV-2 antigens. ^25^ The likely underestimation of seropositivity ^106^ suggests that far more people than officially reported are likely to have had SARS-CoV-2 infections which are unaccounted for. Such a phenomenon would lend support to our suggestion that some populations in Africa may exhibit a blunted, pro-inflammatory adaptive response to SARS-CoV-2 infection. Currently, ongoing works across the 16 administrative regions of Ghana report a SARS-CoV-2 antibody seroprevalence range of approximately 26–57 % as of October 2021 (unpublished work from Rockefeller Foundation Grant Number: 2021 HTH 006).

Post-acute sequelae of COVID-19 lends credence to the suggestion that SARS-CoV-2 infection alters the inflammatory state. Alongside efforts to procure vaccines, therefore, it is just as important that Africa appreciates the potential impact of the apparently common but asymptomatic infections which likely have resulted in widespread natural inoculation. It is necessary to understand what changes in inflammatory state occur post SARS-CoV-2 infection in our populations, to help to characterize and preempt the effects on public health.

## Data availability

No data are associated with this article.

## Authors’ contributions

Daniel Oduro-Mensah: Conceptualization, Writing – original draft, Writing – review and editing. Ebenezer Oduro-Mensah: Writing – original draft, Writing – review and editing. Peter Quashie: Writing – review and editing. Gordon Akanzuwine Awandare: Writing – review and editing. Laud Kenneth Okine: Writing – original draft, Writing – review and editing.
