# Supplementary material for: Explaining the unexpected COVID-19 trends and potential impact across Africa
Source: F1000Res. 2022 Nov 21;10:1177. Originally published 2021 Nov 22. [Version 2] doi: 10.12688/f1000research.74363.2 (PMC9763772; doi:10.12688/f1000research.74363.2)
Supplement: Peer review history

# Peer review: Reviewer response for version 2

**DOI:** 10.5256/f1000research.138619.r157093

**Published:** 2022-12-19

**Authors:** Kurnia Fitri Jamil

**Affiliations:** 1 Universiti Kebangsaan Malaysia, Kuala Lumpur, Malaysia

The suggestions that I can give to the manuscript with the title "Explaining the unexpected COVID-19 trends and potential impact across Africa", are:

1. There are some grammatical errors in the text. Authors need to re-examine the manuscript.
2. I suggest displaying the figure (figure 1), in a more attractive form because the figure that appears, is too often used. There is a lot of information in the manuscript that can be displayed as a figure, making it more interesting. I suggest adding a figure that separates incidence and mortality information.
3. In my opinion, data on the prevalence and specificity in Africa needs to be discussed and studied, which has an impact on the emergence of unexpected trends from a COVID-19.
4. It is better if the authors can provide more updates or additional facts that can be obtained from this study compared to what already been published in the literature, and also present data from other African countries.
5. Reference: correct or cite in full reference numbers 1, 2, 3, 4, 5, 6, 7, 9, 10, 11, 12, 17, 18, 19, 22, 26, 37, 43, 44, 56, 64, 70, 71, 73, 75, 90, 113.

Is the case presented with sufficient detail to be useful for teaching or other practitioners?

Yes

Is the work clearly and accurately presented and does it cite the current literature?

Yes

If applicable, is the statistical analysis and its interpretation appropriate?

Yes

Are all the source data underlying the results available to ensure full reproducibility?

Yes

Are the conclusions drawn adequately supported by the results?

Yes

Is the background of the case’s history and progression described in sufficient detail?

Yes

Reviewer Expertise:

Internist; Tropical Diseases and Infectious Consultant; Professor in Internal Medicine

I confirm that I have read this submission and believe that I have an appropriate level of expertise to confirm that it is of an acceptable scientific standard.

# Peer review: Reviewer response for version 2

**DOI:** 10.5256/f1000research.138619.r156238

**Published:** 2022-11-28

**Authors:** Sankha S. Chakrabarti

**Affiliations:** 1 Department of Geriatric Medicine, Institute of Medical Sciences, Banaras Hindu University, Varanasi, Uttar Pradesh, India

The authors have addressed the concerns raised, although there is still scope for clarity in the final conclusion.

Is the case presented with sufficient detail to be useful for teaching or other practitioners?

Partly

Is the work clearly and accurately presented and does it cite the current literature?

Partly

If applicable, is the statistical analysis and its interpretation appropriate?

Not applicable

Are all the source data underlying the results available to ensure full reproducibility?

Partly

Are the conclusions drawn adequately supported by the results?

Partly

Is the background of the case’s history and progression described in sufficient detail?

Yes

Reviewer Expertise:

COVID vaccines, Geriatric pharmacovigilance, Geriatric neuropsychiatry

I confirm that I have read this submission and believe that I have an appropriate level of expertise to confirm that it is of an acceptable scientific standard.

# Peer review: Reviewer response for version 1

**DOI:** 10.5256/f1000research.78107.r146899

**Published:** 2022-08-30

**Authors:** Sankha S. Chakrabarti

**Affiliations:** 1 Department of Geriatric Medicine, Institute of Medical Sciences, Banaras Hindu University, Varanasi, Uttar Pradesh, India

This is an interesting hypothesis article. There are some minor grammatical errors. Please correct those.

- Example: *"From December 2020 when emerging SARS-CoV-2 variants fueled second and third waves of COVID-19 across the globe ^6^^–^^11^ from December 2020, A"* - Here, *"From December 2020"* is repeated twice. Go through the entire text and check carefully.

My other suggestions are:

 1. Mention low vaccine coverage as a possible reason. Though it may seem unusual, the data for most countries with high vaccination rates such as Israel or South Korea show rising cases post-vaccination and even after boosters. 

 2. The preferred abbreviation for alanine aminotransferase is ALT. Please correct.

 3. The part about obesity and low reactogenicity of vaccines in the elderly as support for the authors' hypothesis is unclear and does not seem convincing. More proof and clarity is needed, or better to delete.

 4. *"Interestingly, the 60+ age group showed the highest seropositivity rate 106. Keeping in mind that the antibody test kit used had shown up to 66 % sensitivity and at least 94 % specificity, ^106^ the report suggested that the observation of low sensitivity of antibody test kits may be due to generally low production of antibodies by infected persons rather than a failure of the kits used. The low sensitivity is interesting, particularly when some studies report significant cross-reactivity between other human coronaviruses antibodies and SARS-CoV-2 antigens. ^25^ The likely underestimation of seropositivity ^106^ suggests that far more people than officially reported are likely to have had SARS-CoV-2 infections which are unaccounted for."* - This part needs to be re-written for clarity. Also, reference 106 at the end of the first sentence is not linked as a reference. Please correct.

 5. The final part (last two paragraphs) are not written in synchrony. The authors have mixed up some important points they wish to make. Please re-write for clarity.

 Overall, the authors start off well but they have not separated the valid points they wish to make clearly in the rest of the article which makes it difficult to understand.

Is the case presented with sufficient detail to be useful for teaching or other practitioners?

Partly

Is the work clearly and accurately presented and does it cite the current literature?

Partly

If applicable, is the statistical analysis and its interpretation appropriate?

Not applicable

Are all the source data underlying the results available to ensure full reproducibility?

Partly

Are the conclusions drawn adequately supported by the results?

Partly

Is the background of the case’s history and progression described in sufficient detail?

Yes

Reviewer Expertise:

COVID vaccines, Geriatric pharmacovigilance, Geriatric neuropsychiatry

I confirm that I have read this submission and believe that I have an appropriate level of expertise to confirm that it is of an acceptable scientific standard, however I have significant reservations, as outlined above.

# Response

**Published:** 2022-09-13

**Authors:** Daniel Oduro-Mensah

**Affiliations:** University of Ghana, Ghana

The authors sincerely appreciate the time and effort the reviewer has spent to make the report better.

 We take note of the reviewer’s comments and respond as follows:

1. We appreciate the reviewer’s suggestion in comment 1 to add to our explanations in the manuscript. However, we believe that it would be a full discussion for another platform. Our thinking about vaccination, as relates to what is presented in this manuscript, has been touched on briefly in the conclusion section.
2. The abbreviation has been corrected to ALT. Thank you very much for the notice in reviewer’s comment 2, that was an oversight.
3. The authors remain committed to the suggestions and inferences the manuscript makes in the section referred to in reviewer’s comment 3. The section has been rewritten and strengthened with further evidence as the reviewer suggested.
4. The section referred to in reviewer’s comment 4 has be rewritten for clarity as suggested by the reviewer.
5. The last two paragraphs have been rewritten as suggested in reviewer’s comment 5. A portion has been broken off to make a conclusion section.
